# Can Urinalysis and Past Medical History of Kidney Stones Predict Urine Antibiotic Resistance?

**DOI:** 10.5811/westjem.2022.4.54872

**Published:** 2022-08-19

**Authors:** Michael Mohseni, Emily C. Craver, Michael G. Heckman, Johnathan M. Sheele

**Affiliations:** * Mayo Clinic, Department of Emergency Medicine, Jacksonville, Florida; † Mayo Clinic, Division of Clinical Trials and Biostatistics, Jacksonville, Florida

## Abstract

**Introduction:**

Urinary tract infections (UTI) are one of the most common infections encountered in the emergency department (ED) with an estimated 2–3 million annual visits. Commonly prescribed antibiotics for UTIs have shown growing rates of resistance. Previous studies lack direction on improving UTI treatment based on the labs available to the bedside clinician.

**Methods:**

We sought to determine if antibiotic resistance in UTIs was related to demographics, urinalysis, and history of renal failure or kidney stones. We conducted an analysis of 892 women ≥18 years of age discharged from the ED with a UTI diagnosis. We assessed predictors of nitrofurantoin resistance, cefazolin resistance, ciprofloxacin resistance, and trimethoprim-sulfamethoxazole resistance using unadjusted and multivariable logistic regression models.

**Results:**

Antibiotic resistance was 13.6% for nitrofurantoin, 11.9% for cefazolin, 12.8% for ciprofloxacin, and 17.1% for trimethoprim-sulfamethoxazole. In multivariable analysis, significant independent associations with an increased likelihood of resistance to nitrofurantoin were observed for less urine blood (OR [per 1 category increase of score] 0.81; P = 0.02); greater mucous (OR [per 1 category increase of score] 1.22; P = 0.02); less specific gravity urine (OR [per 1 category increase] 0.87; P = 0.04), and presence of any history of kidney stones (OR 3.24; P = 0.01). There were no significant predictors for cefazolin resistance (all P ≥0.06); age was the only significant predictor of ciprofloxacin resistance (OR per 10 year increase] 1.10, P = 0.05), and lower specific gravity urine was significantly associated with an increased risk of resistance to trimethoprim- sulfamethoxazole (OR [per 1 category increase] 0.88, P = 0.04).

**Conclusion:**

Women with any history of kidney stones may have bacteriuria resistant to nitrofurantoin, suggesting that providers might consider alternative antibiotic therapies in this scenario.

Urinary tract infections (UTI) are one of the most common infections encountered in the emergency department (ED) with an estimated 2–3 million annual visits.^1^ Widespread rapid antibiotic sensitivity testing is not available during the ED clinical visit; so antibiotics may be prescribed for which the bacteriuria is resistant. Given the difficulty in predicting the proper antimicrobial sensitivity in the setting of emerging and increasingly resistant bacteria, treatment failures may occur.^2^ Commonly prescribed antibiotics for UTIs, including trimethoprim-sulfamethoxazole, fluoroquinolones, and beta-lactams, have all shown growing rates of resistance.^3^ Previous studies have attempted to elucidate general characteristics of antimicrobial resistance of bacteria^4^–^7^ but lack clear direction on improving successful UTI treatment based on the limited laboratory data available to the bedside clinician.

We sought to determine whether nitrofurantoin, cefazolin, ciprofloxacin, and trimethoprim-sulfamethoxazole resistance could be predicted based on triage and demographic data, urinalysis results, and past histories of renal failure/dialysis or kidney stones. We conducted an analysis of an existing dataset of ED patient encounters ≥18 years of age from a single healthcare system between April 18, 2014–March 7, 2017. We examined 892 women discharged from the ED with a UTI based on their discharge *International Classification of Diseases* (ICD) code and who had a positive urine culture (≥10,000 colony forming units per milliliter (CFU/mL) (CFU/mL bacteria in monoculture). Women were considered pregnant if they had a pregnancy-related ICD code or a positive pregnancy test.

We assessed predictors of nitrofurantoin resistance, cefazolin resistance, ciprofloxacin resistance, and trimethoprim-sulfamethoxazole resistance using unadjusted and multivariable logistic regression models. Odds ratios (OR) and 95% confidence intervals (CI) were estimated and are interpreted as the multiplicative increase in the odds of antibiotic resistance for the given antibiotic. Multivariable models were adjusted for any variable with a *P*-value <0.10 in the unadjusted analysis for the given antibiotic resistance outcome (and also had <10% missing data). *P*-values less than 0.05 were considered statistically significant. We performed analyses using R Statistical Software version 4.0.3 (R Foundation for Statistical Computing, Vienna, Austria).

A summary of patient characteristics is shown in Table 1. Median age was 49 years, and 53.3% of the patients were White. Antibiotic resistance was 13.6% for nitrofurantoin, 11.9% for cefazolin, 12.8% for ciprofloxacin, and 17.1% for trimethoprim-sulfamethoxazole. An evaluation of predictors of resistance to nitrofurantoin is provided in Table 2. In multivariable analysis (adjusting for urine blood, mucous, white blood cell clumps, and a history of kidney stones), significant independent associations with resistance to nitrofurantoin were observed for urine blood (OR 0.81; *P* = 0.016); mucous (OR 1.22; *P* = 0.019); specific gravity urine (OR 0.87; *P* = 0.044), and any history of kidney stones (OR 3.24; *P* = 0.013).

Associations of antibiotic resistance for cefazolin, ciprofloxacin, and trimethoprim-sulfamethoxazole are shown in Supplements 1–3. In multivariable analysis, there were no significant predictors for cefazolin resistance (all *P* ≥0.056); age was the only significant predictor of ciprofloxacin resistance (OR 1.10, *P* = 0.048), and specific gravity urine was significantly associated with resistance to trimethoprim-sulfamethoxazole (OR 0.88, *P* = 0.035). For patients resistant to nitrofurantoin, we estimated the proportion who were resistant to our other antibiotics and found that antibiotic resistance was lowest for trimethoprim-sulfamethoxazole (9.6%, 11/115), then ciprofloxacin (14.7%, 17/116), and finally cefazolin (22.6%, 26/115).

One of the risk factors for nitrofurantoin resistance in our study based on multivariable analysis was a history of kidney stones (OR 3.24). Our findings support previous studies finding a higher likelihood of resistant pathogens in patients with a history of nephrolithiasis.^8^–^9^ The *Proteeae* group of bacteria (*Proteus*, *Morganella morganii*, and *Providencia*) are known to produce urease and are associated with kidney stones.^10^ The *Proteeae* group has inherent resistance to nitrofurantoin,^11^ which could explain our findings although our study did not examine which bacteria were growing in patients’ culture or determine whether kidney stones were diagnosed during the current encounter. The clinical significance of our findings remains unclear, but 29.2% (7/24) of women with any history of kidney stones had bacteriuria resistant to nitrofurantoin compared to 13.1% (109/829) for those women without stones. Age ≥65 years was associated with ciprofloxacin resistance, which is consistent with the findings of our study.^12^

The results of this study suggest female UTI patients with any history of kidney stones may have increased rates of treatment failure with nitrofurantoin. Furthermore, in our analysis, antibiotic resistance was lowest with trimethoprim-sulfamethoxazole in those cases of observed nitrofurantoin resistance.

## Supplementary Information



## Figures and Tables

**Table 1:** Summary of patients characteristics in 892 women analyzed..

Variable	N	Median (minimum, maximum) or No. (%) of patients
Age (years)	892	49 (18, 103)
Race	889	
White		474 (53.3%)
Black		405 (45.6%)
Other		10 (1.1%)
Marital status	890	
Single		423 (47.5%)
Married		259 (29.1%)
Other		208 (23.4%)
Primary care doctor	892	378 (42.4%)
Emergency severity index	869	
1		1 (0.1%)
2		34 (3.9%)
3		568 (65.4%)
4		262 (30.1%)
5		4 (0.5%)
Urine specimen source	698	
Clean catheter/voided urine		631 (90.4%)
Straight catheter or urine from new bladder catheter		41 (5.9%)
Bladder catheter not known to be new		25 (3.6%)
Suprapubic catheter		1 (0.1%)
Amorphous crystals urine (positive)	885	62 (7.0%)
Bacteria urine score	886	
0		117 (13.2%)
1		255 (28.8%)
2		159 (17.9%)
3		151 (17.0%)
4		204 (23.0%)
Bilirubin urine score	885	
0		852 (96.3%)
1		11 (1.2%)
2		17 (1.9%)
3		5 (0.6%)
Blood urine score	880	
0		256 (29.1%)
1		189 (21.5%)
2		154 (17.5%)
3		281 (31.9%)
Glucose urine (positive)	886	63 (7.1%)
Ketones urine (positive)	885	115 (13.0%)
Leukocyte esterase urine score	873	
0		76 (8.7%)
1		144 (16.5%)
2		104 (11.9%)
3		549 (62.9%)
Mucous urine score	885	
0		634 (71.6%)
1		133 (15.0%)
2		43 (4.9%)
3		39 (4.4%)
4		36 (4.1%)
Nitrite urine (positive)	885	328 (37.1%)
Urine pH	887	6 (5, 9)
Protein urine (positive)	887	557 (62.8%)
Red blood cells	882	11 (0, 100)
Specific gravity urine	887	
1.000 to 1.004		31 (3.5%)
1.005 to 1.009		227 (25.6%)
1.010 to 1.014		187 (21.1%)
1.015 to 1.019		162 (18.3%)
1.020 to 1.024		128 (14.4%)
1.025 to 1.029		96 (10.8%)
1.030 to 1.034		48 (5.4%)
≥ 1.035		8 (0.9%)
Trichomonas urine (positive)	885	4 (0.5%)
Urobilinogen urine (≥2)	887	173 (19.5%)
White blood cell clumps urine (present)	882	205 (23.2%)
White blood cells	877	36 (0, 100)
Yeast in urine (positive)	885	20 (2.3%)
Pregnant	892	12 (1.3%)
History of renal failure or dialysis	892	21 (2.4%)
History of kidney stones	892	28 (3.1%)
Resistance to cefazolin	831	99 (11.9%)
Resistance to ciprofloxacin	892	114 (12.8%)
Resistance to nitrofurantoin	853	116 (13.6%)
Resistance to trimethoprim-sulfamethoxazole	859	147 (17.1%)

**Table 2:** Evaluation of predictors of resistance to nitrofurantoin.

Variable	N	Unadjusted analysis		Multivariable analysis	
	
		OR (95% CI)	P-value	OR (95% CI)	P-value
Age (10 year increase)	853	1.03 (0.95, 1.12)	0.47	1.05 (0.95, 1.15)	0.34
Race (non-White)	850	1.24 (0.84, 1.84)	0.28	1.16 (0.77, 1.75)	0.49
Marital status	851	Overall test of difference: P=0.49		Overall test of difference: P=0.43	
Single		1.00 (reference)	N/A	1.00 (reference)	N/A
Married		0.90 (0.55, 1.43)	0.65	0.96 (0.58, 1.56)	0.87
Other		1.23 (0.76, 1.97)	0.38	1.34 (0.81, 2.19)	0.25
Primary care doctor	853	1.01 (0.68, 1.50)	0.94	1.02 (0.68, 1.53)	0.91
Emergency severity index (1 unit increase)	830	0.86 (0.59, 1.23)	0.41	0.94 (0.65, 1.36)	0.74
Urine specimen source (non-clean catch/void urine)	670	1.42 (0.68, 2.74)	0.32	1.31 (0.62, 2.58)	0.45
Amorphous crystals urine (positive)	846	1.56 (0.75, 3.00)	0.21	1.53 (0.72, 3.03)	0.24
Bacteria urine score (1 category increase)	847	0.99 (0.86, 1.14)	0.85	0.97 (0.84, 1.12)	0.66
Bilirubin urine score (1 category increase)	846	0.76 (0.34, 1.33)	0.42	0.74 (0.32, 1.30)	0.37
Blood urine score (1 category increase)	842	0.82 (0.69, 0.96)	0.02	0.81 (0.69, 0.96)	0.02
Glucose urine (positive)	848	1.12 (0.50, 2.24)	0.76	1.12 (0.50, 2.27)	0.77
Ketones urine (positive)	846	0.68 (0.33, 1.25)	0.24	0.60 (0.29, 1.12)	0.13
Leukocyte esterase urine score (1 category increase)	836	0.98 (0.81, 1.19)	0.81	1.10 (0.90, 1.36)	0.37
Mucous urine score (1 category increase)	846	1.22 (1.03, 1.44)	0.02	1.22 (1.03, 1.43)	0.02
Nitrite urine (positive)	846	0.73 (0.47, 1.10)	0.13	0.68 (0.44, 1.04)	0.08
Urine pH (1 unit increase)	848	1.07 (0.86, 1.32)	0.53	1.08 (0.87, 1.34)	0.46
Protein urine (positive)	848	0.79 (0.53, 1.19)	0.26	0.99 (0.64, 1.55)	0.98
Red blood cells (10 unit increase)	843	0.96 (0.91, 1.02)	0.23	1.01 (0.94, 1.09)	0.76
Specific gravity urine (1 category increase)	848	0.93 (0.82, 1.05)	0.26	0.87 (0.76, 0.99)	0.04
Urobilinogen urine (≥2)	848	0.95 (0.56, 1.53)	0.83	0.82 (0.48, 1.35)	0.45
White blood cell clumps urine (present)	843	0.64 (0.38, 1.05)	0.09	0.69 (0.40, 1.13)	0.15
White blood cells (10 unit increase)	838	0.99 (0.94, 1.04)	0.69	1.02 (0.96, 1.08)	0.56
Yeast in urine (positive)	846	1.11 (0.26, 3.38)	0.87	1.12 (0.25, 3.52)	0.87
Pregnant	853	0.63 (0.03, 3.35)	0.66	0.58 (0.03, 3.18)	0.61
History of renal failure or dialysis	853	0.70 (0.11, 2.47)	0.64	0.73 (0.11, 2.60)	0.67
History of kidney stones	853	2.72 (1.03, 6.46)	0.03	3.24 (1.21, 7.90)	0.01

ORs are interpreted as the multiplicative increase in the odds of resistance to nitrofurantoin for each increase given in parenthesis (continuous variables) or presence of the given characteristic (categorical variables). Multivariable models were adjusted for all variables with a p-value <0.10 in unadjusted analysis (blood urine score, mucous urine score, WBC clumps urine, and history of kidney stones). The “Overall test of difference” that is provided for marital status tests whether there is any difference in resistance to nitrofurantoin between the three marital status categories.

*OR*, odds ratio; *CI*, confidence interval.
