# Supplementary material for: Can Urinalysis and Past Medical History of Kidney Stones Predict Urine Antibiotic Resistance?
Source: West J Emerg Med. 2022 Aug 19;23(5):613–7. doi: 10.5811/westjem.2022.4.54872 (PMC9541996; doi:10.5811/westjem.2022.4.54872)
Supplement: Supplementary file 1 [file wjem-23-613-s001.docx]

**Supplement 1. Evaluation of predictors of resistance to cefazolin**

|  |  | Unadjusted analysis | | Multivariable analysis | |
| --- | --- | --- | --- | --- | --- |
| Variable | N | OR (95% CI) | P-value | OR (95% CI) | P-value |
| Age (10 year increase) | 831 | 1.06 (0.96, 1.16) | 0.23 | 1.06 (0.96, 1.17) | 0.24 |
| Race (non-White) | 828 | 0.64 (0.41, 0.99) | 0.045 | 0.65 (0.42, 1.01) | 0.056 |
| Marital status | 829 | Overall test of difference: P=0.14 | | Overall test of difference: P=0.26 | |
| Single |  | 1.00 (reference) | N/A | 1.00 (reference) | N/A |
| Married |  | 1.42 (0.86, 2.35) | 0.17 | 1.27 (0.73, 2.18) | 0.39 |
| Other |  | 1.64 (0.97, 2.75) | 0.060 | 1.59 (0.91, 2.75) | 0.10 |
| Primary care doctor | 831 | 1.42 (0.93, 2.17) | 0.10 | 1.39 (0.90, 2.15) | 0.14 |
| Emergency severity index (1 unit increase) | 809 | 0.91 (0.61, 1.34) | 0.63 | 0.86 (0.57, 1.27) | 0.44 |
| Urine specimen source (non-clean catch/void urine) | 652 | 1.55 (0.71, 3.07) | 0.24 | 1.43 (0.65, 2.88) | 0.35 |
| Amorphous crystals urine (positive) | 824 | 1.06 (0.43, 2.27) | 0.88 | 1.20 (0.48, 2.62) | 0.67 |
| Bacteria urine score (1 category increase) | 825 | 1.00 (0.85, 1.16) | 0.96 | 1.00 (0.85, 1.16) | 0.96 |
| Bilirubin urine score (1 category increase) | 824 | 1.48 (0.92, 2.22) | 0.078 | 1.29 (0.79, 1.97) | 0.26 |
| Blood urine score (1 category increase) | 820 | 0.91 (0.76, 1.08) | 0.28 | 0.89 (0.75, 1.07) | 0.22 |
| Glucose urine (positive) | 826 | 1.57 (0.73, 3.10) | 0.21 | 1.69 (0.78, 3.37) | 0.15 |
| Ketones urine (positive) | 824 | 0.76 (0.36, 1.44) | 0.42 | 0.58 (0.26, 1.16) | 0.15 |
| Leukocyte esterase urine score (1 category increase) | 813 | 0.96 (0.78, 1.18) | 0.66 | 0.98 (0.80, 1.21) | 0.85 |
| Mucous urine score (1 category increase) | 824 | 0.87 (0.67, 1.08) | 0.23 | 0.86 (0.66, 1.08) | 0.21 |
| Nitrite urine (positive) | 824 | 0.86 (0.55, 1.33) | 0.50 | 0.82 (0.52, 1.28) | 0.39 |
| Urine pH (1 unit increase) | 826 | 0.95 (0.75, 1.21) | 0.70 | 1.02 (0.78, 1.31) | 0.90 |
| Protein urine (positive) | 826 | 0.95 (0.62, 1.48) | 0.82 | 0.82 (0.52, 1.30) | 0.39 |
| Red blood cells (10 unit increase) | 821 | 1.00 (0.94, 1.06) | 0.97 | 1.00 (0.94, 1.06) | 0.99 |
| Specific gravity urine (1 category increase) | 826 | 1.13 (1.00, 1.28) | 0.057 | 1.13 (0.99, 1.28) | 0.067 |
| Urobilinogen urine (≥2) | 826 | 1.29 (0.77, 2.10) | 0.32 | 1.18 (0.67, 1.98) | 0.56 |
| White blood cell clumps urine (present) | 821 | 0.93 (0.55, 1.51) | 0.77 | 0.90 (0.52, 1.50) | 0.70 |
| White blood cells (10 unit increase) | 816 | 1.04 (0.98, 1.10) | 0.16 | 1.04 (0.99, 1.11) | 0.14 |
| Yeast in urine (positive) | 824 | 2.16 (0.60, 6.18) | 0.18 | 2.05 (0.57, 5.93) | 0.22 |
| Pregnant | 831 | 0.82 (0.04, 4.43) | 0.85 | 0.89 (0.05, 4.86) | 0.91 |
| History of renal failure or dialysis | 831 | 0.87 (0.14, 3.09) | 0.85 | 0.92 (0.14, 3.31) | 0.91 |
| History of kidney stones | 831 | N/A^1^ | 0.098 | N/A | N/A |
| OR=odds ratio; CI=confidence interval. ORs are interpreted as the multiplicative increase in the odds of resistance to nitrofurantoin for each increase given in parenthesis (continuous variables) or presence of the given characteristic (categorical variables). Multivariable models were adjusted for all variables with a p-value <0.10 in unadjusted analysis (race, bilirubin urine score, and specific gravity urine). The “Overall test of difference” that is provided for marital status tests whether there is any difference in resistance to cefazolin between the three marital status categories. ^1^ Logistic regression was not possible for history of kidney stones owing to the presence of a zero cell count; the p-value results from Fisher’s exact test, and this variable was not included in the multivariable analysis despite a p-value <0.10. | | | | | |

**Supplement 2. Evaluation of predictors of resistance to ciprofloxacin**

|  |  | Unadjusted analysis | | Multivariable analysis | |
| --- | --- | --- | --- | --- | --- |
| Variable | N | OR (95% CI) | P-value | OR (95% CI) | P-value |
| Age (10 year increase) | 892 | 1.15 (1.05, 1.25) | 0.002 | 1.10 (1.00, 1.21) | 0.048 |
| Race (non-White) | 889 | 0.66 (0.44, 0.98) | 0.041 | 0.78 (0.50, 1.19) | 0.25 |
| Marital status | 890 | Overall test of difference: P=0.25 | | Overall test of difference: P=0.70 | |
| Single |  | 1.00 (reference) | N/A | 1.00 (reference) | N/A |
| Married |  | 1.21 (0.75, 1.93) | 0.43 | 0.78 (0.44, 1.38) | 0.40 |
| Other |  | 1.51 (0.93, 2.43) | 0.093 | 0.83 (0.43, 1.59) | 0.58 |
| Primary care doctor | 892 | 1.07 (0.72, 1.59) | 0.73 | 0.91 (0.60, 1.38) | 0.67 |
| Emergency severity index (1 unit increase) | 869 | 0.62 (0.42, 0.90) | 0.012 | 0.72 (0.48, 1.06) | 0.10 |
| Urine specimen source (non-clean catch/void urine) | 698 | 2.35 (1.22, 4.31) | 0.008 | 1.74 (0.85, 3.42) | 0.12 |
| Amorphous crystals urine (positive) | 885 | 1.17 (0.53, 2.34) | 0.67 | 1.27 (0.56, 2.61) | 0.54 |
| Bacteria urine score (1 category increase) | 886 | 1.16 (1.00, 1.34) | 0.049 | 1.10 (0.95, 1.28) | 0.20 |
| Bilirubin urine score (1 category increase) | 885 | 1.10 (0.62, 1.72) | 0.71 | 1.10 (0.62, 1.74) | 0.70 |
| Blood urine score (1 category increase) | 880 | 0.87 (0.74, 1.03) | 0.11 | 0.94 (0.78, 1.12) | 0.46 |
| Glucose urine (positive) | 886 | 1.51 (0.73, 2.89) | 0.24 | 1.47 (0.70, 2.84) | 0.28 |
| Ketones urine (positive) | 885 | 1.22 (0.68, 2.08) | 0.49 | 1.13 (0.62, 1.96) | 0.67 |
| Leukocyte esterase urine score (1 category increase) | 873 | 0.96 (0.79, 1.16) | 0.65 | 0.95 (0.79, 1.16) | 0.62 |
| Mucous urine score (1 category increase) | 885 | 0.93 (0.75, 1.13) | 0.48 | 1.05 (0.84, 1.28) | 0.65 |
| Nitrite urine (positive) | 885 | 1.01 (0.66, 1.51) | 0.98 | 0.89 (0.57, 1.37) | 0.60 |
| Urine pH (1 unit increase) | 887 | 1.02 (0.81, 1.26) | 0.87 | 1.02 (0.82, 1.27) | 0.83 |
| Protein urine (positive) | 887 | 0.78 (0.52, 1.16) | 0.22 | 0.86 (0.57, 1.31) | 0.49 |
| Red blood cells (10 unit increase) | 882 | 0.97 (0.91, 1.02) | 0.25 | 1.00 (0.94, 1.06) | 1.00 |
| Specific gravity urine (1 category increase) | 887 | 0.93 (0.82, 1.05) | 0.23 | 0.97 (0.85, 1.10) | 0.59 |
| Urobilinogen urine (≥2) | 887 | 1.00 (0.59, 1.61) | 1.00 | 1.16 (0.68, 1.90) | 0.57 |
| White blood cell clumps urine (present) | 882 | 0.99 (0.61, 1.55) | 0.95 | 0.97 (0.59, 1.54) | 0.89 |
| White blood cells (10 unit increase) | 877 | 0.98 (0.93, 1.03) | 0.36 | 0.99 (0.93, 1.04) | 0.60 |
| Yeast in urine (positive) | 885 | 1.21 (0.28, 3.68) | 0.76 | 1.43 (0.33, 4.48) | 0.58 |
| Pregnant | 892 | N/A^1^ | 0.38 | N/A | N/A |
| History of renal failure or dialysis | 892 | 1.14 (0.26, 3.44) | 0.83 | 0.68 (0.15, 2.14) | 0.55 |
| History of kidney stones | 892 | 1.91 (0.69, 4.54) | 0.17 | 1.80 (0.65, 4.36) | 0.22 |
| OR=odds ratio; CI=confidence interval. ORs are interpreted as the multiplicative increase in the odds of resistance to nitrofurantoin for each increase given in parenthesis (continuous variables) or presence of the given characteristic (categorical variables). Multivariable models were adjusted for all variables with a p-value <0.10 in unadjusted analysis (age, race, emergency severity index, and bacteria urine score). Urine specimen source was not included in the multivariable analysis due to the extent of missing data for this variable. The “Overall test of difference” that is provided for marital status tests whether there is any difference in resistance to ciprofloxacin between the three marital status categories. ^1^ Logistic regression for pregnant was not possible owing to the presence of a zero cell count; the p-value results from Fisher’s exact test. | | | | | |

**Supplement 3. Evaluation of predictors of resistance to trimethoprim-sulfamethoxazole**

|  |  | Unadjusted analysis | | Multivariable analysis | |
| --- | --- | --- | --- | --- | --- |
| Variable | N | OR (95% CI) | P-value | OR (95% CI) | P-value |
| Age (10 year increase) | 859 | 1.02 (0.94, 1.10) | 0.67 | 1.00 (0.92, 1.08) | 0.98 |
| Race (non-White) | 856 | 0.79 (0.55, 1.13) | 0.20 | 0.84 (0.58, 1.20) | 0.34 |
| Marital status | 857 | Overall test of difference: P=0.17 | | Overall test of difference: P=0.28 | |
| Single |  | 1.00 (reference) | N/A | 1.00 (reference) | N/A |
| Married |  | 1.08 (0.70, 1.66) | 0.72 | 1.01 (0.65, 1.55) | 0.98 |
| Other |  | 1.51 (0.98, 2.32) | 0.060 | 1.40 (0.90, 2.16) | 0.13 |
| Primary care doctor | 859 | 1.11 (0.78, 1.59) | 0.56 | 1.11 (0.77, 1.59) | 0.56 |
| Emergency severity index (1 unit increase) | 836 | 1.15 (0.83, 1.60) | 0.40 | 1.20 (0.85, 1.69) | 0.29 |
| Urine specimen source (non-clean catch/void urine) | 673 | 1.00 (0.48, 1.91) | 1.00 | 1.03 (0.50, 1.98) | 0.92 |
| Amorphous crystals urine (positive) | 852 | 0.64 (0.26, 1.35) | 0.28 | 0.68 (0.28, 1.44) | 0.35 |
| Bacteria urine score (1 category increase) | 853 | 1.02 (0.90, 1.17) | 0.72 | 1.03 (0.91, 1.18) | 0.62 |
| Bilirubin urine score (1 category increase) | 852 | 0.76 (0.36, 1.28) | 0.37 | 0.82 (0.39, 1.39) | 0.53 |
| Blood urine score (1 category increase) | 847 | 0.97 (0.84, 1.12) | 0.67 | 0.97 (0.84, 1.13) | 0.70 |
| Glucose urine (positive) | 853 | 1.08 (0.52, 2.05) | 0.82 | 1.08 (0.52, 2.06) | 0.83 |
| Ketones urine (positive) | 852 | 1.26 (0.74, 2.05) | 0.37 | 1.34 (0.79, 2.21) | 0.26 |
| Leukocyte esterase urine score (1 category increase) | 840 | 1.05 (0.88, 1.26) | 0.61 | 1.03 (0.87, 1.24) | 0.73 |
| Mucous urine score (1 category increase) | 852 | 0.86 (0.70, 1.04) | 0.14 | 0.91 (0.74, 1.10) | 0.36 |
| Nitrite urine (positive) | 852 | 0.90 (0.62, 1.30) | 0.58 | 0.92 (0.63, 1.33) | 0.66 |
| Urine pH (1 unit increase) | 854 | 1.01 (0.82, 1.23) | 0.94 | 0.94 (0.77, 1.16) | 0.58 |
| Protein urine (positive) | 854 | 1.13 (0.78, 1.66) | 0.51 | 1.26 (0.86, 1.86) | 0.25 |
| Red blood cells (10 unit increase) | 849 | 0.99 (0.94, 1.04) | 0.75 | 1.00 (0.95, 1.05) | 0.93 |
| Specific gravity urine (1 category increase) | 854 | 0.88 (0.79, 0.99) | 0.035 | 0.88 (0.79, 0.99) | 0.035 |
| Urobilinogen urine (≥2) | 854 | 1.15 (0.73, 1.76) | 0.54 | 1.23 (0.78, 1.89) | 0.37 |
| White blood cell clumps urine (present) | 849 | 1.18 (0.78, 1.76) | 0.43 | 1.17 (0.77, 1.76) | 0.45 |
| White blood cells (10 unit increase) | 844 | 1.01 (0.97, 1.06) | 0.61 | 1.01 (0.97, 1.06) | 0.56 |
| Yeast in urine (positive) | 852 | 1.40 (0.39, 3.98) | 0.55 | 1.47 (0.41, 4.21) | 0.50 |
| Pregnant | 859 | 0.48 (0.03, 2.54) | 0.49 | 0.51 (0.03, 2.73) | 0.53 |
| History of renal failure or dialysis | 859 | 0.85 (0.20, 2.58) | 0.80 | 0.82 (0.19, 2.49) | 0.76 |
| History of kidney stones | 859 | 0.40 (0.06, 1.35) | 0.21 | 0.39 (0.06, 1.32) | 0.20 |
| OR=odds ratio; CI=confidence interval. ORs are interpreted as the multiplicative increase in the odds of resistance to nitrofurantoin for each increase given in parenthesis (continuous variables) or presence of the given characteristic (categorical variables). Multivariable models were adjusted for all variables with a p-value <0.10 in unadjusted analysis (specific gravity urine). The “Overall test of difference” that is provided for marital status tests whether there is any difference in resistance to trimethoprim-sulfamethoxazole between the three marital status categories. | | | | | |
